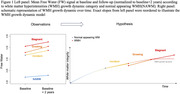# Free water is a sensitive predictor of future white matter lesions

**DOI:** 10.1002/alz.093370

**Published:** 2025-01-09

**Authors:** Pauline Maillard, Evan Fletcher, Oliver Martinez, Baljeet Singh, Francisco Sandoval, Audrey P. Fan, Sarah Tomaszewski Farias, Charles Decarli

**Affiliations:** ^1^ University of California, Davis, Davis, CA USA; ^2^ University of California Davis, Davis, CA USA; ^3^ University of California, Davis, Sacramento, CA USA

## Abstract

**Background:**

Cerebrovascular disease features as the most common comorbidity in older individuals with dementia. White matter (WM) injury identified through white matter hyperintensities (WMH) on FLAIR represents evident late‐stage pathophysiological manifestations of CVD. This study aims to assess the role of baseline microstructural WM integrity, using free water (FW), a measure from diffusion tensor imaging (DTI), in predicting WMH growth dynamic.

**Methods:**

Our study includes 271 individuals (age: 76±7, 60% female) from the UCD ADRC longitudinal cohort who underwent an MRI exam at two different date (mean time±SD: 2.7±1.2 years). We tracked individual lesions between baseline and follow‐up. Briefly, for each participant, distinct WMH region were labeled on their baseline and follow‐up scans, coregistered into the FSL template space. All lesions were then placed into three categories: 1) baseline WMH that do not get larger over time were labeled stagnant WMH, 2) baseline WMH that grow larger over time were labeled growing WMH, and (3) WMH identified only on follow‐up scans were labeled noncontiguous incident WMH. DTI‐derived mean signal of Free Water (FW) was computed for each individual WMH region at baseline, as well as within the normal appearing WM (NAWM). Using a linear mixed‐effect model, we investigated differences in baseline FW according each WMH tissue category and NAWM. Analyses were adjusted for age and sex.

**Results:**

At baseline, FW signal was found to be lower within NAWM as compared with growing WMH (b=‐0.11, pvalue< 0.001, see Figure 1) and stagnant WMH (b=‐0.13, pvalue< 0.001), and also with incident WMH (b=‐0.096, pvalue< 0.001) suggesting that WM tissue where incident WMH are to emerge over time is already injured as compared to normal WM. Baseline FW for stagnant WMH was significantly higher than for growing (b=0.027, pvalue< 0.001) and incident WMH (b=0.038, pvalue< 0.001). Baseline FW for incident WMH was not found to be significantly different from baseline FW in growing WMH (b=0.011, pvalue=0.078).

**Conclusion:**

Our results suggest that local FW measures predict the future incidence as well as the growth dynamic of lesions over time and emphasize the sensitivity of FW as a biomarker of early CVD‐related WM injury.